# Neuropathy predicts early renal impairment in diabetes mellitus—the almost forgotten historical evidence. In memoriam Professor Göran Sundkvist

**DOI:** 10.3389/fcdhc.2026.1875311

**Published:** 2026-06-09

**Authors:** Peter Kempler, Dóra Marietta Balogh, Viktor J. Horváth, Adrienn Menyhárt, Manfredi Rizzo, Solomon Tesfaye, Vincenza Spallone

**Affiliations:** 1 Department of Medicine and Oncology, Semmelweis University, Budapest, Hungary; 2 University Hospital of Palermo, University of Palermo, Palermo, Italy; 3 Diabetes Research Unit, Sheffield Teaching Hospitals and the University of Sheffield, Sheffield, United Kingdom; 4 Department of Systems Medicine, University of Rome Tor Vergata, Rome, Italy

**Keywords:** autonomic nervous system, diabetic nephropathy, diabetic neuropathy, interoception, kidney innervation

## Abstract

A recent cross-sectional study found an association between peripheral neuropathy, assessed by the Toronto Clinical Neuropathy Score (TCNS), and diabetic nephropathy based on microalbuminuria, in individuals with well-controlled type 2 diabetes and preserved renal function. These findings echo the pioneering work of Göran Sundkvist, who in 1992, at the 2nd annual meeting of the Diabetic Neuropathy Study Group of the EASD (Neurodiab), presented a prospective study with a follow-up period of 10–11 years in type 1 diabetes, showing that cardiac autonomic neuropathy (CAN) predicts renal function decline. Since its publication in 1993, several subsequent longitudinal studies have confirmed this predictive value, supporting the role of CAN as a progression promoter of diabetic nephropathy. The link between these two microangiopathic complications extends beyond shared pathogenetic mechanisms driven by hyperglycaemia and other cardiovascular risk factors. It reflects a complex bidirectional neural network connecting brain and kidney through the autonomic nervous system. Renal innervation expresses not only neural control by the central and autonomic nervous systems, but also the interoceptive ability to modulate sympathetic nervous system activity and to play a role in cardiovascular control. This multiorgan network is disrupted in diabetes and represents a potential therapeutic target. Göran Sundkvist, one of the first members of the Neurodiab, deserves to be remembered for his contribution: he passed away in 2006 after the closure of the Neurodiab meeting he organized in Ystad (Sweden).

## Introduction

Zaki et al. recently reported on 122 patients with type 2 diabetes (T2DM) (HbA1c <7%, eGFR >90 ml/min/1.73m^2^) whether there is an association between early diabetic peripheral neuropathy and subclinical diabetic nephropathy (1). Neuropathy was determined using the Toronto Clinical Neuropathy Score (TCNS), evaluating symptoms and signs of neuropathy; the urinary albumin-to-creatinine ratio (UACR) was used for assessment of nephropathy. Although neither electrophysiological methods nor small-fiber function tests were applied, the TCNS served as a validated, reliable, and even cost-effective tool for the assessment of distal symmetrical polyneuropathy.

## The association between diabetic neuropathy and nephropathy

Neuropathy and nephropathy are classical microvascular complications of diabetes mellitus, and both are associated with poor prognosis. We agree with the conclusion of the authors that early diabetic peripheral neuropathy measured by the TCNS is significantly associated with early nephropathy characterized by higher UACR in well-controlled T2DM patients with preserved renal function.

The study’s limitations are appropriately listed by the authors. Nonetheless, due to the cross-sectional design of the study, the paper’s title may not be valid; in some patients, increase in UACR might precede early neuropathy. However, in most patients, neuropathy develops earlier than nephropathy, although depending on diagnostic methods as well. The authors correctly quote up-to-date references discussing the connection between diabetic neuropathy and nephropathy; the two oldest citations are from 2005.

## Pioneering studies by Goran Sundkvist

It is worth noting that 34 years ago the Professor Göran Sundkvist gave a lecture (2) at the second meeting of Neurodiab (Diabetic Neuropathy Study Group of the EASD) in Bratislava (Slovakia) and reported that cardiac autonomic neuropathy (CAN) predicts deterioration in glomerular filtration rate in patients with insulin-dependent diabetes mellitus. His findings were severely criticized during the meeting; however, they were subsequently published in Diabetes Care (2). In a prospective study with a follow-up period of 10–11 years, 20 of 35 patients developed CAN assessed by heart rate responses to deep breathing and tilt test. Among these patients, the decrease of glomerular filtration rate (GFR) was fourfold compared to those without CAN. The mean GFR among subjects with CAN was in the normal range even at the end of the study.

## Discussion

There are several differences between the studies of Zaki et al. (1) and Sundkvist et al. (2). The study of Zaki et al. was cross-sectional, and clinical peripheral neuropathy was assessed among T2DM patients. Sundkvist et aal. evaluated CAN in patients with type 1 diabetes (T1DM) as part of a 10-11-year-long follow-up study (2). The study commenced around 1980, and at that time, T1DM was much more in the focus of clinical and scientific interest compared to T2DM. It should be noted that T1DM subjects examined by Sundkvist et al. (3) had signs of peripheral neuropathy as well, especially autonomic nerve dysfunction (2). Zaki et al. (1) have not reported on autonomic dysfunction but given the close relationship between distal symmetrical polyneuropathy and CAN, they are likely to have had CAN also. In the context of a cross-sectional study, one is able to determine concurrent validity relating early neuropathy with nephropathy, but the predictive validity of neuropathy for incident nephropathy in T2DM can only be ascertained in a well-designed prospective study such as the one by Sundkvist et al. in T1DM all those years ago (2). Since that first study, 16 of 18 longitudinal studies found measures of CAN as independent predictors of diabetic nephropathy progression, mainly decline in eGFR, founding the concept of CAN as a progression promoter of this condition (4).

The mechanisms behind this relation had not been fully understood, but research identified in the same years or shortly after changes associated with CAN as potential mechanisms as increased nocturnal blood pressure, loss of neurally mediated adjustment in renal haemodynamics, abnormal circadian profile of sympatho-vagal activity, EPO dysregulation leading to anaemia (4), and more recently CAN-driven inflammation and oxidative stress (5). Thus, Sundkvist’s work pioneered research into the role of autonomic control of kidney in diabetes and its dysfunction in diabetic nephropathy.

**Figure 1:**
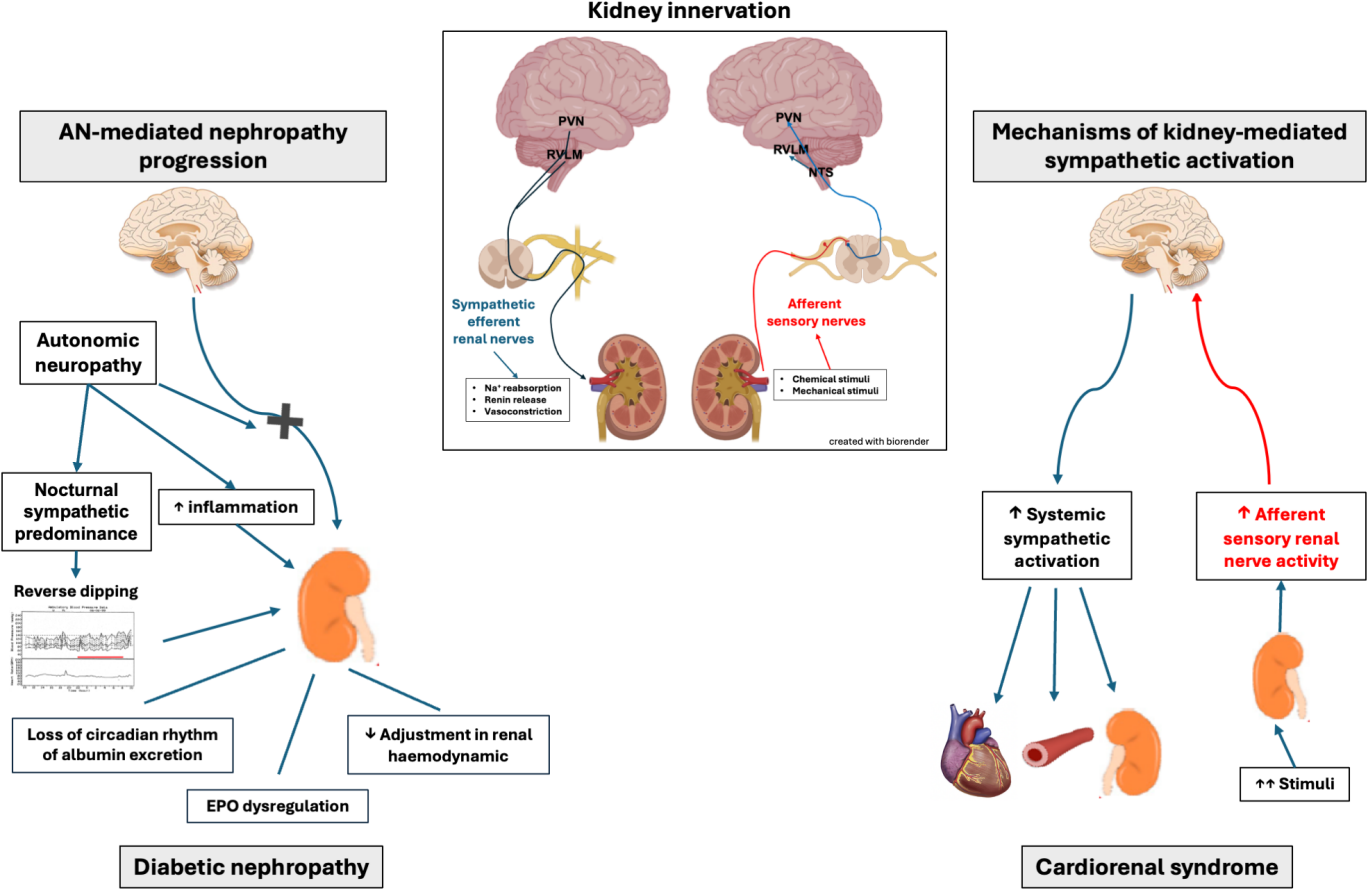
Kidney innervation (center). Kidney receives sympathetic efferent renal nerves from the rostral ventral lateral medulla (RVLM) in the brainstem and the hypothalamic paraventricular nucleus (PVN), which promote tubular sodium reabsorption, renin release, and vasoconstriction. Afferent sensory nerves project to the nucleus tractus solitarius (NTS) in the brainstem and then to PVN. When activated by mechanical and chemical stimuli, they transmit to the brain to increase sympathetic activity. AN-mediated nephropathy progression (left side). Diabetic autonomic neuropathy may accelerate diabetic nephropathy by disrupting the circadian rhythm of sympatho-vagal activity, inducing reverse dipping and changes in albuminuria daily profile, impairing neural regulation of renal haemodynamics, and affecting erythropoietin. The sympatho-vagal imbalance exerts a proinflammatory effect worsening kidney disease (5). Kidney-mediated sympathetic activation (right side). In kidney disease models, hypoxia triggers a chemoreceptor-driven renorenal reflex centered in PVN, resulting in systemic sympathetic activation (7). Drug-induced modulation of intrarenal stimuli might reverse this effect. AN, autonomic neuropathy; EPO, erythropoietin; NTS, nucleus tractus solitarius; PVN, paraventricular nucleus; RVLM, rostral ventral lateral medulla.

Moreover, Sundkvist’s paper opened to the relevance of kidney innervation in cardio-renal diseases, which received increasing support in the last decades by the clinical trials on renal denervation for resistant hypertension and those using SGLT2i whose favourable cardiovascular outcome was partially attributed to an autonomic mechanism (6). Animal studies have provided insights into the function of renal innervation in its two components of efferent sympathetic and afferent sensory nerves, positioning the kidney as both a target of autonomic control but also an interoceptive organ able to communicate with the brain and regulate for its part sympathetic activity to other organs (7) (**Figure 1**). This new knowledge has the potentiality of translation into novel treatment of cardiorenal diseases (7).

It is worth remembering that Professor Göran Sundkvist, who worked at the Department of Medicine and Clinical Physiology, University of Lund, Malmö, Sweden, was one of the original, pioneer researchers in the field of neuropathy. Professor Sundkvist was one of the first members of Neurodiab; he served in the Executive Committee from 1998 to 2003, and moreover he organized two Neurodiab annual meetings: Stockholm, Sweden, in 1995 and Ystad, Sweden, in 2006. He passed away on Friday 15 September at his office, 2 days after the closure of the meeting in Ystad 20 years ago. In 2007, the Young Investigators Award for Clinical Science of the Neurodiab has been named after Göran Sundkvist, a pioneering researcher in Diabetes.
