# Correction: Neuropathy predicts early renal impairment in diabetes mellitus—the almost forgotten historical evidence. In memoriam Professor Göran Sundkvist

**DOI:** 10.3389/fcdhc.2026.1918820

**Published:** 2026-07-15

**Authors:** Peter Kempler, Dóra Marietta Balogh, Viktor J. Horváth, Adrienn Menyhárt, Manfredi Rizzo, Solomon Tesfaye, Vincenza Spallone

**Affiliations:** 1 Department of Medicine and Oncology, Semmelweis University, Budapest, Hungary; 2 University Hospital of Palermo, University of Palermo, Palermo, Italy; 3 Diabetes Research Unit, Sheffield Teaching Hospitals and the University of Sheffield, Sheffield, United Kingdom; 4 Department of Systems Medicine, University of Rome Tor Vergata, Rome, Italy

**Keywords:** autonomic nervous system, diabetic nephropathy, diabetic neuropathy, interoception, kidney innervation

The article type was erroneously given as “Opinion”. The correct article type is “Perspective”.

The missing **Abstract** was added:

“A recent cross-sectional study found an association between peripheral neuropathy, assessed by the Toronto Clinical Neuropathy Score (TCNS), and diabetic nephropathy based on microalbuminuria, in individuals with well-controlled type 2 diabetes and preserved renal function. These findings echo the pioneering work of Göran Sundkvist, who in 1992, at the 2nd annual meeting of the Diabetic Neuropathy Study Group of the EASD (Neurodiab), presented a prospective study with a follow-up period of 10–11 years in type 1 diabetes, showing that cardiac autonomic neuropathy (CAN) predicts renal function decline. Since its publication in 1993, several subsequent longitudinal studies have confirmed this predictive value, supporting the role of CAN as a progression promoter of diabetic nephropathy.”

“The link between these two microangiopathic complications extends beyond shared pathogenetic mechanisms driven by hyperglycaemia and other cardiovascular risk factors. It reflects a complex bidirectional neural network connecting brain and kidney through the autonomic nervous system. Renal innervation expresses not only neural control by the central and autonomic nervous systems, but also the interoceptive ability to modulate sympathetic nervous system activity and to play a role in cardiovascular control. This multiorgan network is disrupted in diabetes and represents a potential therapeutic target.”

“Göran Sundkvist, one of the first members of the Neurodiab, deserves to be remembered for his contribution: he passed away in 2006 after the closure of the Neurodiab meeting he organized in Ystad (Sweden).”

The original version of this article has been updated.

